# Individual and area-level determinants associated with C-reactive protein as a marker of cardiometabolic risk among adults: Results from the German National Health Interview and Examination Survey 2008-2011

**DOI:** 10.1371/journal.pone.0211774

**Published:** 2019-02-08

**Authors:** Henriette Steppuhn, Detlef Laußmann, Jens Baumert, Lars Kroll, Thomas Lampert, Dietrich Plaß, Christa Scheidt-Nave, Christin Heidemann

**Affiliations:** 1 Department of Epidemiology and Health Monitoring, Robert Koch Institute, Berlin, Germany; 2 German Center for Diabetes Research (DZD), München-Neuherberg, Germany; 3 Department of Environmental Hygiene, German Environment Agency, Berlin, Germany; University of Glasgow, UNITED KINGDOM

## Abstract

**Background:**

High-sensitivity C-reactive protein (hsCRP) is a sensitive biomarker of systemic inflammation and is related to the development and progression of cardiometabolic diseases. Beyond individual-level determinants, characteristics of the residential physical and social environment are increasingly recognized as contextual determinants of systemic inflammation and cardiometabolic risks. Based on a large nationwide sample of adults in Germany, we analyzed the cross-sectional association of hsCRP with residential environment characteristics. We specifically asked whether these associations are observed independent of determinants at the individual level.

**Methods:**

Data on serum hsCRP levels and individual sociodemographic, behavioral, and anthropometric characteristics were available from the German Health Interview and Examination Survey for Adults (2008–2011). Area-level variables included, firstly, the predefined German Index of Socioeconomic Deprivation (GISD) derived from the INKAR (indicators and maps on spatial and urban development in Germany and Europe) database and, secondly, population-weighted annual average concentration of particulate matter (PM_10_) in ambient air provided by the German Environment Agency. Associations with log-transformed hsCRP levels were analyzed using random-intercept multi-level linear regression models including 6,768 participants aged 18–79 years nested in 162 municipalities.

**Results:**

No statistically significant association of PM_10_ exposure with hsCRP was observed. However, adults residing in municipalities with high compared to those with low social deprivation showed significantly elevated hsCRP levels (change in geometric mean 13.5%, 95%CI 3.2%-24.7%) after adjusting for age and sex. The observed relationship was independent of individual-level educational status. Further adjustment for smoking, sports activity, and abdominal obesity appeared to markedly reduce the association between area-level social deprivation and hsCRP, whereas all individual-level variables contributed significantly to the model.

**Conclusions:**

Area-level social deprivation is associated with higher systemic inflammation and the potentially mediating role of modifiable risk factors needs further elucidation. Identifying and assessing the source-specific harmful components of ambient air pollution in population-based studies remains challenging.

## Introduction

C-reactive protein (CRP) is an acute phase protein which has been widely used as a non-specific biomarker of systemic inflammation [1–3]. Chronic low-grade systemic inflammation as measured by slightly to moderately elevated high sensitivity CRP (hsCRP) has gained clinical attention based on epidemiological studies indicating its role as an independent predictor of type 2 diabetes mellitus (T2DM) [4, 5], cardiovascular morbidity, and cardiovascular as well as all-cause mortality [2, 6–9]. In clinical practice, hsCRP measurement may have an additive value to CVD risk assessment and therapy decisions among patient subgroups [7, 8, 10–12]. At the population level, hsCRP has gained significance as a highly standardized integral marker of health risks associated with cardiometabolic and other aging-related health conditions [2, 6, 9, 13, 14].

Elevated hsCRP levels are consistently related to a variety of modifiable risk factors of cardiometabolic and other major chronic diseases [2, 8, 15, 16]. This is particularly true for abdominal obesity, smoking, and low physical activity [15, 17–23]. In addition, there is mounting evidence that nutritional components and dietary patterns have an impact on systemic inflammation [24–28]. Besides, increased psychosocial stress may also contribute to systemic inflammation as measured by hsCRP [15, 16, 29, 30]. Social differences in modifiable risk factors may, in part, explain variations of hsCRP consistently observed according to individual-level educational attainment [15, 31–35].

Systemic inflammation may also be in the pathway of the association between characteristics of the residential physical and social environment with cardiometabolic diseases [34, 36–49]. Results from recent population-based studies suggest that exposure to ambient air pollution from traffic sources may result in increased systemic inflammation [37, 38, 46]. Further to this point, living in more deprived residential environments has been related to increased cardiometabolic risk [39–42, 47, 48, 50]. However, while area-level deprivation has been linked to T2DM risk based on observational and interventional studies [39–42, 47, 50], corresponding evidence on a potentially underlying association with systemic inflammation is scarce and inconsistent [34, 45, 48, 51].

Against this background, we assessed individual and contextual determinants of hsCRP as an established non-specific biomarker of systemic inflammation using data from a nationally representative sample of adults aged 18 to 79 years in Germany. We specifically asked whether exposure to ambient particulate air pollution and social deprivation at residence is associated with hsCRP and whether these associations are observed independent of individual sociodemographic, behavioral, and anthropometric determinants.

## Material and methods

### Study design and study population

The present analysis is based on individual-level data from the German Health Interview and Examination Survey for Adults (DEGS1, 2008–2011) which is an observational study with a mixed design allowing for cross-sectional and longitudinal analyses [52–54]. The target population of DEGS1 comprised the residential, non-institutionalized adult population aged 18–79 years in Germany. First-time participants were selected at random drawn from local population registries based on a two-stage cluster sampling design [53, 54]. In addition, DEGS1 included a panel component, in which individuals who had previously participated in the earlier German National Health Interview and Examination Survey (GNHIES98, 1997–1999) were re-contacted and invited to also participate in DEGS1 [53, 54]. The net sample of 7,987 individuals allows for representative cross-sectional analyses for the age range of 18–79 years and includes 4,192 newly recruited persons with a response rate of 42% and 3,795 previously participating persons with a response rate of 64% [53, 54]. Among these, a total of 7,115 participants completed both the interview and examination part [53, 54].

The study protocol of the DEGS1 survey was approved by the Federal and State Commissioners for Data Protection and the local ethics committee, Charité - Universitätsmedizin Berlin ethics committee (ethics approval application document number: EA2/047/08) [53, 54]. The implementation of the survey conforms to the principles of the Helsinki Declaration [53, 54]. Prior to the interview and examination, participants provided written informed consent [53, 54].

### Outcome measure

HsCRP was measured by nephelometry with high sensitivity by using a latex enhanced nephelometric assay (Siemens BN ProSpec Analyzer). The standards of the assays were prepared by Siemens Healthcare Diagnostic Products (Inc., Marburg, Germany) and measurement was performed by the central epidemiological laboratory unit at the Robert Koch Institute accredited according to DIN EN 15189 und DIN EN 17025. Blood samples were processed within one hour and serum was stored at -40°C until analysis in the central laboratory unit. The lowest reportable hsCRP level was 0.15 mg/L. For hsCRP levels outside the detection range which amounted to 4.9% of eligible subjects, hsCRP values were imputed as follows [55, 56]. HsCRP levels were log-transformed for normality of right skewed distributed measurement values. Mean and standard deviation of the underlying unrestricted lognormal distribution were estimated by using the R packages truncdist and fitdistrplus [56–58]. Based on the estimated distribution parameters, a random sample of lognormally distributed hsCRP values was generated for imputation of hsCRP values outside the detection range by using the R package fitdistrplus [56, 58].

### Individual-level sociodemographic, behavioral, and anthropometric characteristics

Information on sociodemographic and behavioral factors was obtained from a self-administered questionnaire. Age was treated as a continuous variable and mean-centered for the analysis. Individual-level educational attainment was classified according to the Comparative Analysis of Social Mobility in Industrial Nations (CASMIN) classification system [59]. Smoking status was assessed as “never smoking”, “former smoking”, “occasional smoking”, “daily smoking”. Sports activity was assessed using five answer categories (“no”, “less than 1 h”, “regularly 1–2 h”,”regularly 2–4 h”, “regularly more than 4 h”) categorized into “less than 2 h per week” and “regularly, ≥2 h per week” [60]. Standardized measurement of waist circumference at the minimal waist of study participants or at the midpoint between the lowest rib and the ileac crest among those with no visible waist was performed by trained staff [61, 62]. Abdominal obesity was defined as a waist circumference greater than 102 cm in men or 88 cm in women [63–65].

### Area-level characteristics

Firstly, area-level deprivation was defined according to the recently published German Index of Socioeconomic Deprivation (GISD) that uses data from the official INKAR (indicators and maps on spatial and urban development) database of regional indicators compiled by Germany’s Federal Institute for Research on Building, Urban Affairs, and Spatial Development (BBSR) [66]. The method used to develop the GISD is described in detail elsewhere [66]. In brief, the index was constructed as a multi-dimensional measure of regional socio-economic deprivation based on the three equally weighted dimensions of education, occupation, and income. The GISD was calculated at different regional levels as of 31.12.2012. For the current analysis, the smallest available geographic unit was considered comprising 4,504 Gemeindeverbände (associations of municipalities). Overall, these municipalities had an average population of 17,878 inhabitants (range; 338–3,375,222 inhabitants) and an average size of 78.4 km^2^ (2.0–891.7 km^2^) in 2012 [66, 67]. The corresponding features of the 162 municipalities included in the DEGS1 study were as follows: average population of 127,306 inhabitants (1,906–3,375,222 inhabitants) and average size of 124.9 km^2^ (16.4–891.7 km^2^). The GISD can be obtained freely from the Github online repository [68] as a three-level variable classified into low (lowest quintile), medium (middle three quintiles) and high (highest quintile) levels of socioeconomic deprivation for the year 2008 [66].

Secondly, we used data on population-weighted annual average concentration of particulate matter of the fraction with less than 10 micrometers in diameter (PM_10_) in ambient air provided by the German environment agency for the year 2009 [69]. These data combine information on measured modelled urban and rural background concentration levels of PM_10_ with information on population density at an 1 km x 1 km resolution. This allows to estimate population-weighted annual average PM_10_ concentration levels per cubic meter at the municipality or national level [69]. For the analyses, PM_10_ concentration was grouped into prespecified exposure categories with an interval size of 5 μg/m^3^ [69] and further dichotomized at a cut-off level of ≥20μg/m^3^ (vs. <20μg/m^3^) according to the PM_10_ guideline value proposed by the World Health Organization [69, 70]. In additional analyses, PM_10_ concentration was also grouped into three levels based on the distribution among study participants (lowest quintile, middle three quintiles, highest quintile).

### Statistical analyses

CRP values were log-transformed to reach an approximately normal distribution and treated as a continuous outcome variable. STATA (StataCorp) software was applied in all the analyses. In order to account for the complex survey design and deviation of the sample from the population structure as of 31 Dec 2010, bivariate analyses were performed by using specific survey weights [53]. The Rao-Scott chi-square test of independence with second order adjustment [71] was applied to globally test for differences in the hsCRP concentration level categories indicating lower (<1 mg/L), average (1–3 mg/L) and higher relative vascular risk (>3 mg/L including subjects with marked elevation of hsCRP above 10 mg/L) [2] by categorical study variables. Differences in hsCRP mean values across study variable categories were tested based on linear regression models for which log-transformed hsCRP values were treated as outcome and geometric mean hsCRP levels were obtained by anti-log transformation of (arithmetic) mean log-transformed hsCRP which gives the geometric mean of hsCRP.

In multivariable linear regression analyses, random-intercept multilevel models were applied in order to assess the association of individual and municipality-level factors with log-transformed hsCRP values as outcome by using the Stata command “meglm”. While accounting for the clustering nature of the sample design, this command did not allow performing additional weighting of results to adjust for deviation of the sample from the population structure as of 31 Dec 2010. Weights-related variables were, however, considered in the multivariable analyses. Effect estimates are presented as the percentage change in geometric mean of the outcome hsCRP across categories of independent variables compared to a reference category, together with 95% confidence interval (CI) using the anti-logs of regression estimates. The percentage change in geometric mean can be interpreted approximately as percentage change in median hsCRP on the original (mg/L) scale due to the approximately lognormal distribution of hsCRP [72]. Besides sex and age, individual and area-level variables statistical significantly associated with hsCRP in bivariate analyses were sequentially included in multivariable regression models as follows: model 1: area-level variables adjusted for sex and age; model 2: further adjusted for educational attainment; model 3: further adjusted for smoking and physical activity; model 4: further adjusted for abdominal obesity. Missing values were excluded from the analyses and complete-case analyses were performed among 6,768 adults nested in 162 municipalities with complete information on all variables of interest. The analyses were repeated after excluding hsCRP values above 10 mg/L (n = 236) as this may indicate a current bacterial infection [2].

## Results

**Fig 1:**
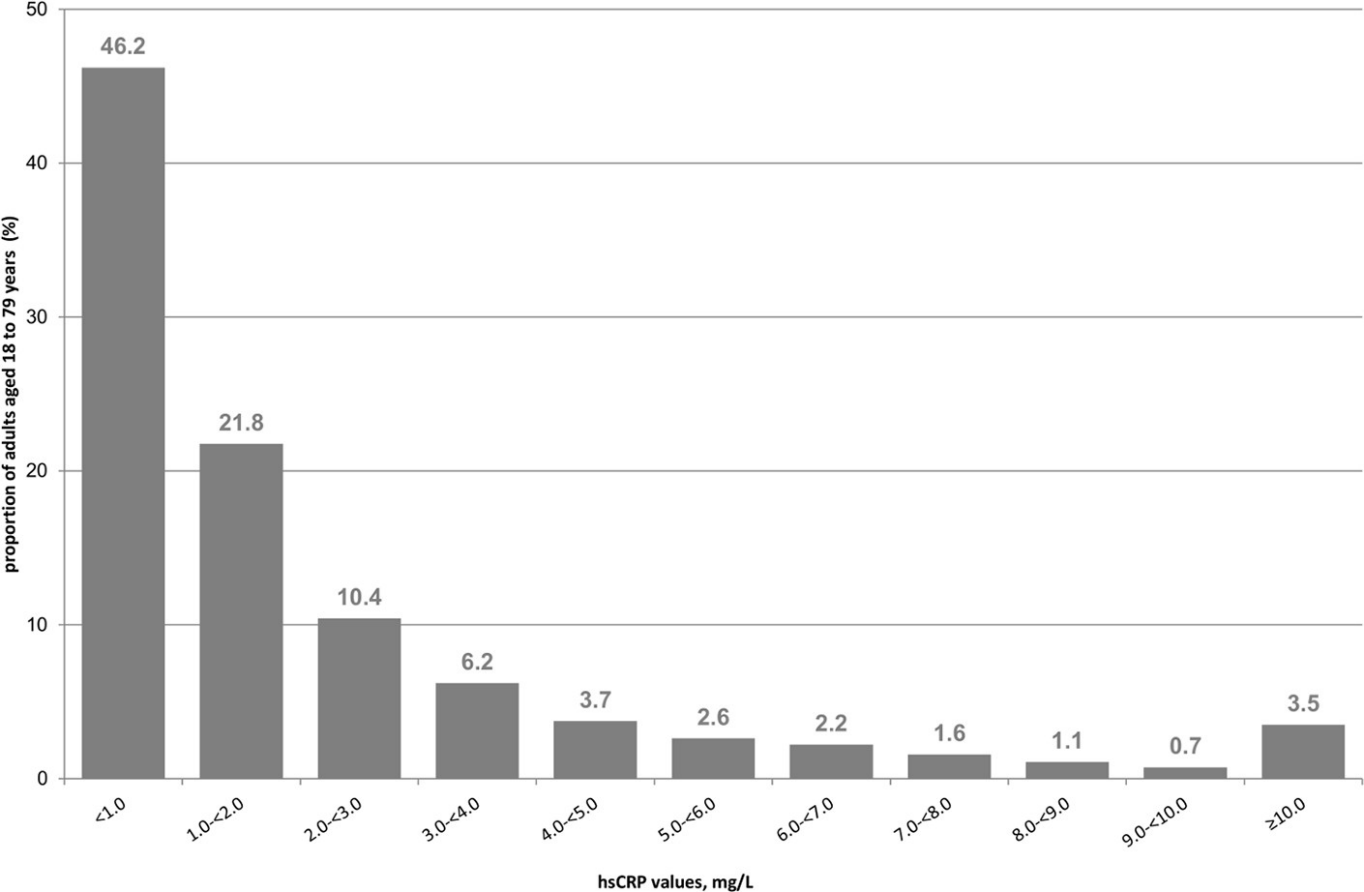
Distribution of hsCRP levels among adults aged 18–79 years (n = 7,006) in the German National Health Interview and Examination Survey (DEGS1, 2008–2011). Percentage, % are weighted to represent the German residential population.

Among adults aged 18 to 79 years, the overall geometric mean of serum-specific hsCRP was 1.15 mg/L (95%-CI; 1.11–1.21). Fig 1 depicts the distribution of hsCRP values in the study population. HsCRP was measured at concentration levels indicating lower CVD risk (hsCRP <1 mg/L) among half of participants (Table 1). HsCRP values indicating average CVD risk (1–3 mg/L) were observed among one third and respective values indicating higher CVD risk (>3 mg/L) among one fifth of participants (Table 1) including 3.4% with marked elevation of hsCRP above 10 mg/L. Descriptive characteristics of study participants nested within 162 municipalities are shown in Table 1.

**Table 1:** Descriptive characteristics of adults 18–79 years (n = 7,006)^§^ in the German National Health Interview and Examination Survey (DEGS1, 2008–11).

Characteristics	n^§^	% (95%-CI)^#^	Mean (SD)^#^
***Individual-level variables***			
**Sex**			
Men	3,366	49.9 (48.3–51.4)	
Women	3,640	50.1 (48.6–51.7)	
**Age, years**			47.41 (16.65)
18–29 years	1,049	18.8 (17.9–19.7)	
30–44 years	1,412	25.1 (23.9–26.3)	
45–64 years	2,729	36.7 (35.3–38.1)	
65–79 years	1,816	19.4 (18.4–20.4)	
**Educational attainment (CASMIN)**			
Low	2,277	37.0 (34.9–39.2)	
Middle	3,370	48.5 (46.8–50.3)	
High	1,314	14.5 (13.0–16.0)	
**Smoking status**			
Never smoking	3,023	41.9 (40.4–43.4)	
Former smoking	2,087	28.2 (27.0–29.5)	
Occasional smoking	411	6.2 (5.5–6.9)	
Daily smoking	1,445	23.7 (22.3–25.3)	
**Sports activity**			
<2h per week	5,104	74.5 (73.0–75.9)	
Regularly, ≥2h per week	1,712	25.5 (24.1–27.0)	
**Abdominal obesity**			
No	4,373	66.2 (64.3–68.1)	
Yes	2,602	33.8 (31.9–35.7)	
***Municipality-level variables***			
**Population-weighted ambient PM**_**10**_ **concentration**			18.54 (3.72)
<10 μg/m^3^	-	-	
≥10-<15 μg/m^3^	1,142	17.9 (12.5–24.9)	
≥15-<20 μg/m^3^	3,469	48.3 (40.5–56.1)	
≥20-<25 μg/m^3^	2,230	31.0 (24.4–38.5)	
≥25-<30 μg/m^3^	165	2.9 (1.2–6.8)	
≥30 μg/m^3^	-	-	
**Social deprivation (GISD)**			
Low	1,330	21.8 (15.9–29.2)	
Middle	3,623	54.5 (46.6–62.1)	
High	2,053	23.7 (18.0–30.6)	
***Outcome***			
**HsCRP concentration**			1.15 (3.36)^&^
<1 mg/L	3,091	46.2 (44.5–47.8)	
1–3 mg/L	2,390	32.3 (30.9–33.6)	
>3 mg/L	1,525	21.6 (20.3–22.9)	

^§^Numbers of observations per variable (n) are unweighted and may vary due to number of missing values for each variable.

^#^Percentage, % (95% confidence intervals; 95%-CI) and mean (standard deviation; SD) are weighted to represent the German residential population and refer to participants providing information for the respective variable of interest. ^&^Geometric mean and standard deviation.

### Bivariate analyses on the associations of individual- and area-level variables with hsCRP

Regarding individual-level variables, higher hsCRP values were significantly associated with age, female sex, low (vs. high) education, current daily and former (vs. never) smoking, lower sports activity and abdominal obesity (Table 2). As for area-level variables, a significantly higher geometric mean of hsCRP values was seen for high vs. low social deprivation (1.32 vs. 1.09 mg/L). One in four participants residing in areas with high social deprivation had a hsCRP levels >3 mg/L, i.e. showed systemic inflammation at a level indicating higher CVD risk, while this proportion amounted to only about one in five participants residing in areas with low social deprivation. Although the highest geometric mean of hsCRP values was found in the highest PM_10_ exposure category (≥25 μg/m^3^), no statistically significant association with population-weighted PM_10_ concentration was observed (Table 2) regardless of the respective exposure operationalization (S1 Table).

**Table 2:** Bivariate associations of individual and area-level variables with hsCRP among adults aged 18–79 years (n = 6,768).

Characteristics	Categories of hsCRP			*P* value*	Geometric mean of hsCRP		*P* value**
	<1 mg/L %	1–3 mg/L %	>3 mg/L %		mg/L		
***Individual-level variables***							
**Total**	46.4	32.3	21.3		1.15		
**Sex**				<0.001			
Men	49.6	33.0	17.4		1.03		<0.001
Women	43.2	31.5	25.3		1.28		
**Age**							
18–29 years	58.7	23.6	17.7	<0.001	0.83		<0.001
30–44 years	54.0	28.2	17.8		0.94		
45–64 years	42.3	35.5	22.3		1.30		
65–79 years	31.6	40.4	28.0		1.65		
**Educational attainment (CASMIN)**							
Low	36.3	35.9	27.8	<0.001	1.47		<0.001
Middle	50.9	30.5	18.7		1.03		
High	55.9	29.6	14.5		0.91		
**Smoking status**							
Never smoking	49.8	31.3	19.0	<0.001	1.06		<0.001
Former smoking	44.4	33.5	22.1		1.18		
Occasional smoking	58.4	26.8	14.8		0.90		
Daily smoking	39.8	34.0	26.2		1.35		
**Sports activity**							
<2h per week	42.7	33.8	23.5	<0.001	1.27		<0.001
Regularly, ≥2h per week	57.1	27.7	15.2		0.85		
**Abdominal obesity**							
No	57.5	28.0	14.5	<0.001	0.86		<0.001
Yes	24.3	40.8	35.0		2.06		
***Municipality-level variables***							
**Population-weighted ambient PM**_**10**_ **concentration**							
<15 μg/m^3^	44.4	33.2	22.4	0.398	1.20		0.394
≥15-<20 μg/m^3^	46.6	33.0	20.4		1.13		
≥20-<25 μg/m^3^	47.6	30.8	21.6		1.13		
≥25 μg/m^3^	42.4	30.0	27.6		1.30		
**Social deprivation (GISD)**							
Low	47.9	31.9	20.2	0.044	1.09		<0.001
Middle	47.5	32.0	20.5		1.10		
High	42.3	33.4	24.3		1.32		

All results are weighted to represent the German residential population.

*P-values obtained from Rao-Scott chi-square test of independence with second order adjustment.

**P-values for change in mean log-transformed hsCRP values obtained from linear regression models.

### Multivariable analyses on the associations of individual- and area-level variables with hsCRP

Adjusting for age and sex, a significant 13.5% change in geometric mean of hsCRP values was observed among adults residing in municipalities with high compared to those with low social deprivation (model 1 in Table 3). Results were not materially changed after further adjusting for individual-level educational attainment (model 2). Additional inclusion of smoking, physical activity (model 3), and abdominal obesity (model 4), however, markedly reduced the association between municipality-level social deprivation and hsCRP which was no longer statistically significant. Regarding individual-level variables, an inverse association between educational attainment and hsCRP was only partly attenuated after adjustment for behavioral and anthropometric factors. Daily smoking, lower sports activity, and abdominal obesity remained consistent determinants of hsCRP in the fully adjusted model (model 4). Similar findings were obtained after excluding individuals with hsCRP values above 10 mg/L (Table 4).

**Table 3:** Multivariable associations of individual and area-level variables with hsCRP among adults aged 18–79 years (n = 6,768).

Characteristics	% change in geometric mean (95% confidence intervals)			
	Model 1	Model 2	Model 3	Model 4
***Individual level***				
Women vs. men	20.4 (13.9–27.2)	18.6 (12.3–25.3)	20.3 (13.8–27.1)	16.3 (10.3–22.7)
Age [years]	1.5 (1.3–1.7)	1.4 (1.2–1.6)	1.5 (1.3–1.7)	0.8 (0.7–1.0)
Middle vs. high education (CASMIN)		22.4 (13.5–32.0)	17.0 (8.5–26.1)	11.8 (4.0–20.2)
Low vs. high education (CASMIN)		44.4 (33.2–56.5)	33.7 (23.3–44.9)	22.2 (13.0–32.1)
Former vs. never smoking			12.7 (5.6–20.4)	7.6 (1.0–14.6)
Occasional vs. never smoking			5.5 ((-6.6)-19.0)	5.8 ((-5.8)-18.9)
Daily smoking vs. never smoking			35 (25.2–45.5)	36.3 (26.7–46.5)
Sports activity <2h vs. ≥2h/week			31.5 (23.4–40.1)	19.9 (12.7–27.5)
Abdominal obesity				98.7 (87.2–110.8)
***Municipality level***				
Middle vs. low deprivation (GISD)	2.6 ((-5.9)-11.8)	1.5 ((-6.9)-10.6)	-0.5 ((-8.6)-8.3)	-3.5 ((-11.2)-5.0)
High vs. low deprivation (GISD)	13.5 (3.2–24.7)	13.2 (3.1–24.4)	8.9 ((-0.7)-19.5)	2.4 ((-6.6)-12.2)

Model 1: area-level deprivation adjusted for sex and age; model 2: further adjusted for educational attainment; model 3: further adjusted for smoking and physical activity; model 4: further adjusted for abdominal obesity.

**Table 4:** Multivariable associations of individual and area-level variables with hsCRP among adults aged 18–79 years and hsCRP≤10 mg/l (n = 6,532).

Characteristics	% change in geometric mean (95% confidence intervals)			
	Model 1	Model 2	Model 3	Model 4
***Individual level***				
Women vs. men	18.6 (12.7–24.8)	17.0 (11.2–23.1)	18.6 (12.7–24.8)	15.6 (10.1–21.5)
Age [years]	1.5 (1.4–1.7)	1.4 (1.3–1.6)	1.5 (1.3–1.6)	0.9 (0.7–1.1)
Middle vs. high education (CASMIN)		21.3 (13.2–30.0)	16.6 (8.8–25.0)	11.9 (4.7–19.7)
Low vs. high education (CASMIN)		39.5 (29.5–50.2)	30.6 (21.3–40.8)	20.5 (12.1–29.6)
Former vs. never smoking			12.6 (6.0–19.5)	7.8 (1.7–14.2)
Occasional vs. never smoking			4.2 ((-6.8)-16.5)	4.7 ((-6.0)-16.6)
Daily smoking vs. never smoking			29.3 (20.6–38.7)	30.5 (22.0–39.7)
Sports activity <2h vs. ≥2h/week			25.6 (18.5–33.1)	15.7 (9.3–22.4)
Abdominal obesity				88.5 (78.4–99.2)
***Municipality level***				
Middle vs. low deprivation (GISD)	-0.1 ((-7.5)-7.9)	-1.1 ((-8.4)-6.9)	-2.7 ((-9.8)-5.0)	-5.4 ((-12.4)-2.2)
High vs. low deprivation (GISD)	9.1 (0.4–18.7)	9.0 (0.2–18.5)	5.5 ((-2.9)-14.6)	-0.3 ((-8.3)-8.4)

Model 1: area-level deprivation adjusted for sex and age; model 2: further adjusted for educational attainment; model 3: further adjusted for smoking and physical activity; model 4: further adjusted for abdominal obesity.

## Discussion

Using a large nationwide sample of the general adult population in Germany, the present study demonstrated that social deprivation as measured by the recently developed GISD was related to higher hsCRP values. A 13.5% change in the geometric mean of hsCRP was observed among adults residing in municipalities with high compared to those with low social deprivation adjusting for age and sex and this difference can be interpreted as a similar change in median hsCRP on the original (mg/L) scale [72]. The association between GISD and systemic inflammation was independent of participants’ educational attainment but appeared to be markedly reduced by inclusion of individual-level information on smoking, physical activity, and abdominal obesity. The present study, therefore, extends results from most recent nationwide investigations demonstrating a consistent correlation between the GISD as an indicator of area-level social deprivation and these behavioral and anthropometric determinants [66]. Although the highest geometric mean of hsCRP was observed in the highest PM_10_ exposure category, there was no statistically significant association of area-level PM_10_ exposure with hsCRP.

### Association of ambient PM_10_ exposure with hsCRP

There are heterogenous results from previous population-based studies on the association between residential exposure to ambient fine particulate matter and systemic inflammation in the general adult population [38, 46, 73, 74]. Inconsistencies in the observed effects have been partly attributed to differences in composition and sources of the total PM mixture, e.g. with respect to traffic-related and metal-rich PM in comparison to other PM mixtures [37, 38, 46, 74]. In line with our findings, one recent meta-analysis of pooled data from 22,561 adults in Europe showed that exposure to several size fractions of PM at residential address in 2008–2011 was not or only inconsistently related to biomarkers of systemic inflammation including hsCRP [37, 38]. The study was based on five European cohorts including the two regional German studies KORA (Cooperative Health Research in the Region of Augsburg) and HNR (Heinz Nixdorf Recall) [38]. However, site-specific analyses of these pooled data from European cohort studies showed a significant association of PM_2.5_ exposure with hsCRP only among HNR participants residing in the Ruhr area of Germany. In particular, a 5 μg/m^3^ increase in PM_2.5_ levels was related to a significant 16.1% increase in geometric mean hsCRP [38]. It was hypothesized that these inconsistent findings might be due to differences in PM composition between study sites particularly with respect to harmful components such as transition metals from traffic sources [37, 38]. Accordingly, a more in-depth analysis of these pooled European cohort data suggested that long-term exposure to transition metals within PM_10_ and PM_2.5_ may be associated with chronic systemic inflammation as measured by hsCRP [37]. In addition to these findings, another investigation based on the HNR data showed that long-term exposure to traffic-specific PM_2.5_ and PM_10_ was found to be more strongly associated with systemic inflammation than total PM_2.5_ and PM_10_ [46].

### Association of social deprivation with hsCRP

In the present analysis, serum hsCRP was associated with indicators of social differences both at the individual and area level. Daily smoking, lower sports activity, and abdominal obesity apparently mediated this relationship in statistical models, especially at the area level. Previous studies likewise indicated a substantial mediating effect of these modifiable risk factors in the cross-sectional and longitudinal association of district or neighborhood-level deprivation with T2DM [41, 42, 50]. However, few population-based studies have investigated on an association of social deprivation with systemic inflammation which might act as an underlying mechanism for area-level differences in cardiometabolic risk.

Analyses on the association of neighborhood deprivation with systemic inflammation among adults have, so far, been available from North America and revealed heterogeneous results depending on the investigated biomarker [34, 45, 48, 51]. In particular interleukin 6 (IL-6), a cytokine which induces the production of CRP and other acute phase reactants and thus represents a more upstream biomarker of the inflammatory response, was found to be associated with neighborhood social status [45, 48]. In contrast, the relationship with hsCRP appeared to be less consistent and more sensitive to adjustment for sociodemographic characteristics or other determinants including behavioral and anthropometric factors [34, 45, 48, 51]. In addition, one study examined the longitudinal associations of neighborhood characteristics with changes in inflammation as measured by IL-6 [48]. In this previous analysis, higher neighborhood deprivation was significantly associated with higher increases in IL-6 over an observation period of 3 to 4 years indicating a potential contribution of systemic inflammation to area-level differences in cardiometabolic risk [48]. This association remained significant even after adjusting for sociodemographic, behavioral, and anthropometric factors [48]. Therefore, other factors resulting from increased psychosocial stress have been discussed to contribute as well.

There are different mechanisms through which characteristics of deprived residential environments might affect behavioral and anthropometric determinants of systemic inflammation. Among these, quality and structure of the built environment, e.g. access to recreational resources and degree of neighborhood walkability, might influence physical activity levels and anthropometric measures [75–77]. Further, lower neighborhood safety associated with area-level deprivation may result in increased inflammation by limiting the possibilities for safe recreation and activity or inducing psychosocial stress [48, 51, 78]. In addition, residential environments might also lead to acute elevations of circulating hsCRP by influencing the risk of obtaining an infection. In order to rule out potential cases with current infections, marked elevations of hsCRP levels (>10 mg/L) would have needed confirmation within 4 weeks [2]. In the present study, we repeated the analyses after excluding participants with hsCRP levels above 10 mg/L and obtained similar results.

### Strengths and limitations

The present study is among the few studies that assessed individual and contextual correlates of low-grade systemic inflammation and adds to the highly limited knowledge on the relationship between residential neighborhood characteristics, modifiable risk factors, and health status. Our analysis is based on data from a large nationally representative study of the non-institutionalized, resident adult population, which extends the generalizability of our findings. There are some limitations to the interpretation of our current findings. At foremost, the cross-sectional study design precludes any temporal or causal inferences. In particular, the interrelationship between area-level deprivation, modifiable individual-level determinants, and hsCRP can only be disentangled in further prospective studies.

In the present study, we used the multi-dimensional GISD which was just recently developed in order to reflect regional socio-economic deprivation [66]. The GISD was chosen because this index has been composed of socioeconomic indicators only. By contrast, indices of multiple deprivation have also considered other indicators that do not permit a clear conceptual distinction between determinants and consequences of diseases [66, 79]. The GISD was derived from official statistics and administrative data that were in part only available at the regional level of districts. It was, thus, also projected at the smaller level of municipalities to allow for more detailed characterization of the spatial units [66, 80]. However, in order to further elucidate the mechanisms underlying area-level social differences in systemic inflammation and cardiometabolic risk, higher resolution spatial data would have been preferable [80]. In addition, other factors such as residential neighborhood cohesion or safety should also be considered [48, 51, 77, 78]. Moreover, due to the use of administrative boundaries the modifiable area unit problem (MAUP) needs to be considered when interpreting the results [81]. This is of particular relevance due to the varying size of the municipalities included in the present analysis [81]. Therefore, a replication of the results using more equally defined regions or geocoded data would be desirable in future studies [81].

Finally, it was not possible to obtain exposure estimates based on modeled air pollution concentrations at the geo-coded residential addresses of survey participants. By contrast, we used data aggregated at the municipality level which combined information on measured and modelled urban and rural background PM_10_ concentration levels and estimated numbers of exposed people in 2009 [69]. This approach allowed for estimating residential ambient PM_10_ exposure in a large nationally representative sample and yielded a distribution of exposed German adults across PM_10_ concentration levels that was highly consistent with results obtained from calculations for Germany overall (S2 Table) [69]. However, the modelled PM_10_ exposure used in the analyses did not include high concentrations found close to areas with increased traffic-related air pollution (hot-spots). Thus, an underestimation of the overall residential exposure can be assumed [69]. Moreover, between and within municipality heterogeneity in composition and sources of the total PM mixture could not be addressed.

## Conclusions

In this large nationally representative study of adults in Germany, serum hsCRP as a marker of systemic inflammation was associated with indicators of social differences both at the individual and the municipality level. Modifiable individual-level determinants of hsCRP, including daily smoking, lower sports activity, and abdominal obesity apparently mediated this relationship in statistical models, especially in the association with area-level social deprivation. Further research is needed to unravel the intricate interrelation between residential neighborhood characteristics, modifiable risk factors, and health status. Such information is urgently needed for effective primary and secondary prevention of major chronic diseases and health capacity building among disadvantaged groups of the population. Regarding the effects of ambient particulate matter pollution on systemic inflammation, identification and assessment of source-specific harmful components in population-based studies remains challenging.

## Supporting information

S1 Table Bivariate associations of differently operationalized population-weighted PM_10_ concentration with hsCRP among adults aged 18–79 years (n = 6,768) (pdf file 229 kb). (PDF)

S2 Table Residential population-weighted ambient PM_10_ concentration levels in 2009 across exposure categories in the DEGS1 study sample of adults aged 18–79 years and in the total population in Germany in 2009 (pdf file 285 kb). (PDF)
