# Supplementary material for: Individual and area-level determinants associated with C-reactive protein as a marker of cardiometabolic risk among adults: Results from the German National Health Interview and Examination Survey 2008-2011
Source: PLoS One. 2019 Feb 8;14(2):e0211774. doi: 10.1371/journal.pone.0211774 (PMC6368296; doi:10.1371/journal.pone.0211774)
Supplement: S1 Table — (PDF) [file pone.0211774.s001.pdf]

S1 Table. Bivariate associations of differently operationalized population-weighted PM_{10} concentration with hsCRP among adults aged 18-79 years (n=6,768).

Population-weighted ambient PM_{10} concentration	Categories of hsCRP <1 mg/L	1-3 mg/L	>3 mg/L	*P* value*	Geometric mean of hsCRP mg/L	*P* value**
**Exposure category**						
<15 µg/m³	44.4	33.2	22.4	0.398	1.20	0.394
≥15-<20 µg/m³	46.6	33.0	20.4		1.13	
≥20-<25 µg/m³	47.6	30.8	21.6		1.13	
≥25 µg/m³	42.4	30.0	27.6		1.30	
<20 µg/m³	46.0	33.1	20.9	0.292	1.15	0.884
≥20 µg/m³	47.2	30.7	22.1		1.14	
Lowest quintile	44.6	31.5	24.0	0.089	1.20	0.227
Middle three quintiles	47.8	32.2	20.0		1.11	
Highest quintile	44.0	33.5	22.5		1.21	

**P*-values obtained from Rao-Scott chi-square test of independence with second order adjustment. ***P*-values for change in mean values obtained from linear regression models.
