# Supplementary material for: Individual and area-level determinants associated with C-reactive protein as a marker of cardiometabolic risk among adults: Results from the German National Health Interview and Examination Survey 2008-2011
Source: PLoS One. 2019 Feb 8;14(2):e0211774. doi: 10.1371/journal.pone.0211774 (PMC6368296; doi:10.1371/journal.pone.0211774)
Supplement: S2 Table — (PDF) [file pone.0211774.s002.pdf]

S2 Table. Residential population-weighted ambient PM_{10} concentration levels in 2009 across exposure categories in the DEGS1 study sample of adults aged 18-79 years and in the total population in Germany in 2009.

Population-weighted ambient PM_{10} concentration	DEGS1 sample			Total population in Germany	
	**%* (95%CI*)**	**Mean**		**%****	**Mean****
**Mean [µg/m³]**		18.5			18.7
**Exposure category**					
Level 1 (<10 µg/m³)	-			-	
Level 2 (≥10-<15 µg/m³)	17.9 (12.5-24.9)			14.9	
Level 3 (≥15-<20 µg/m³)	48.3 (40.5-56.1)			50.3	
Level 4 (≥20-<25 µg/m³)	31.0 (24.4-38.5)			29.9	
Level 5 (≥25-<30 µg/m³)	2.9 (1.2-6.8)			4.7	
Level 6 (≥30-<35 µg/m³)	-			0.2	
Level 7 (≥35-<40 µg/m³)	-			-	
Level 8 (≥40 µg/m³)	-			-	
**Level ≥4 (≥20 µg/m³)**	33.8 (27.0-41.4)			34.8	

*Results are weighted to represent the German residential population. **Data source: German Environment Agency.
